# Supplementary material for: CD8 lymphocytes mitigate HIV-1 persistence in lymph node follicular helper T cells during hyperacute-treated infection
Source: Nat Commun. 2022 Jul 12;13:4041. doi: 10.1038/s41467-022-31692-8 (PMC9279299; doi:10.1038/s41467-022-31692-8)
Supplement: Supplementary file 1 — Supplementary Information [file 41467_2022_31692_MOESM1_ESM.pdf]

Supplementary Figure 1

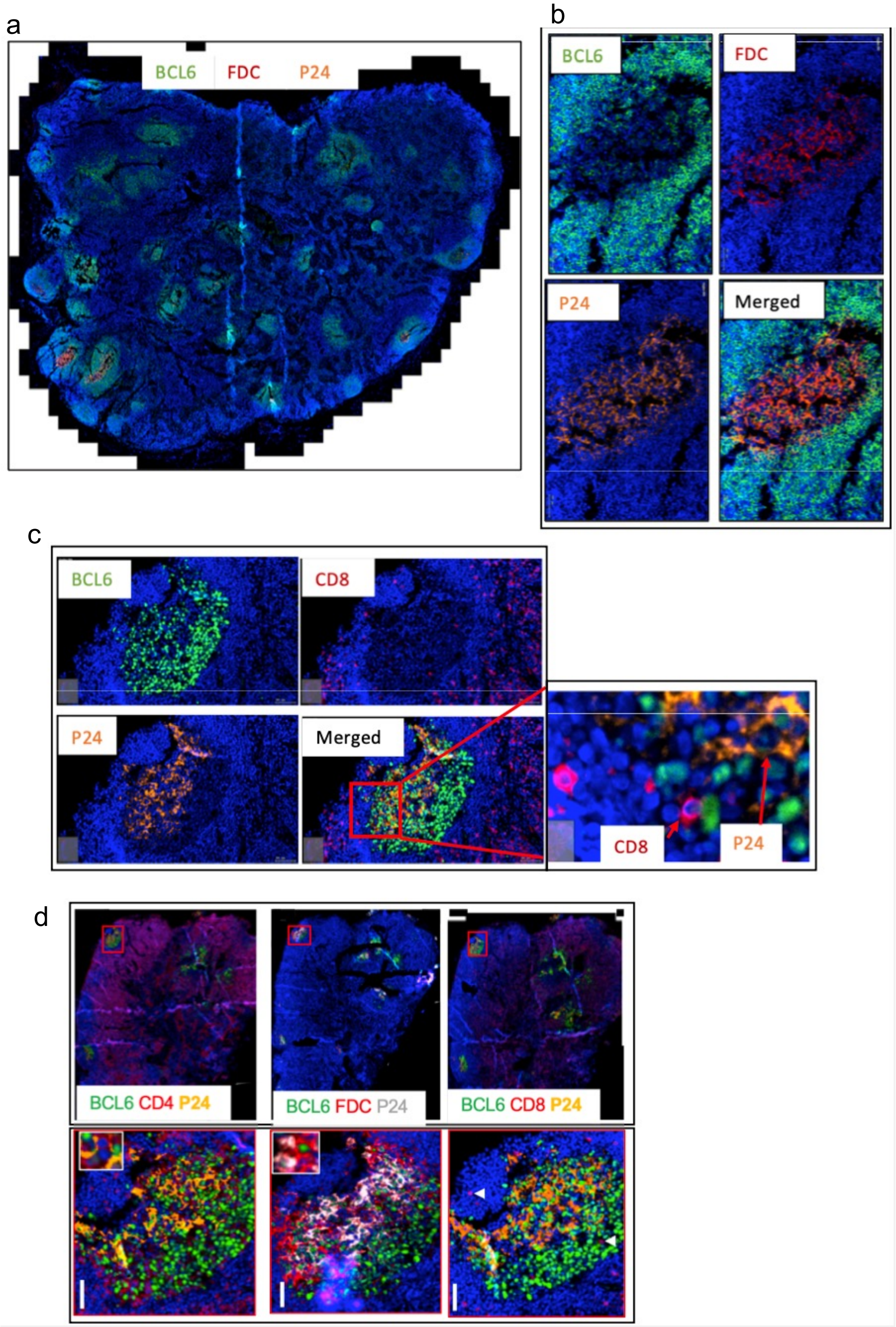

Supplementary Figure 1

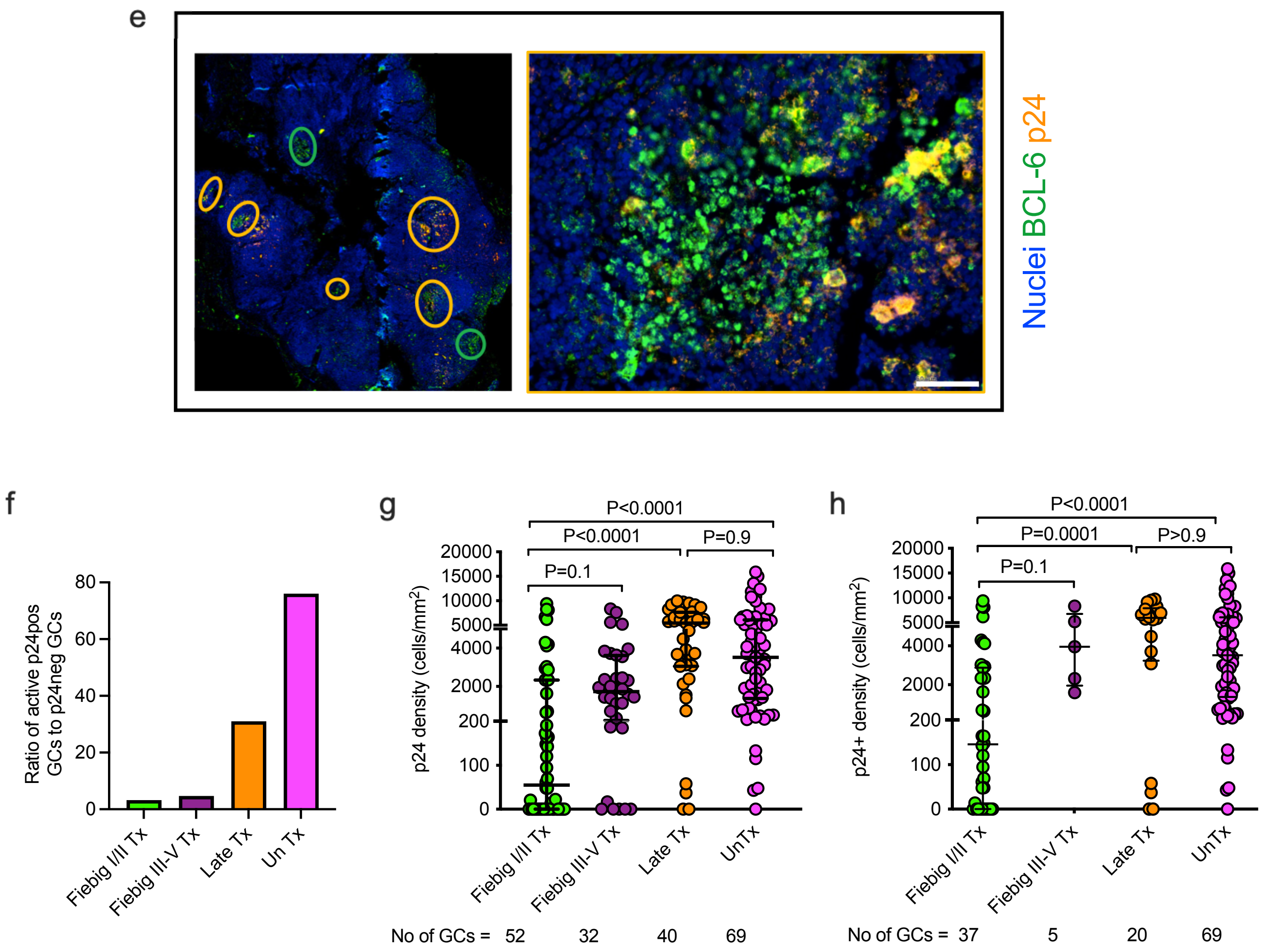

Supplementary Figure 1

i

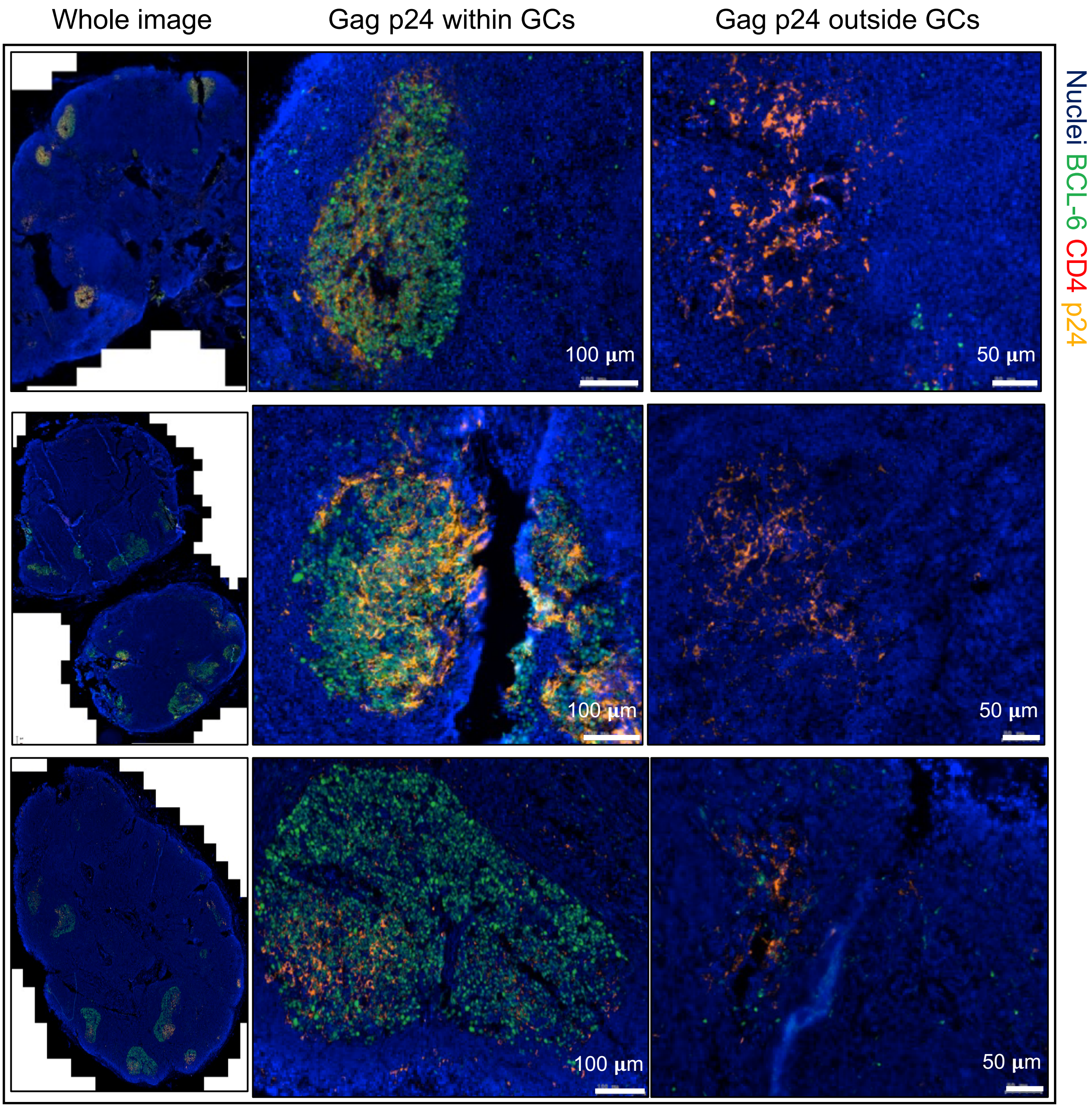

Supplementary Figure 1: HIV Gag p24 staining co-localizes with Follicular dendritic cells and BCL-6 but not with CD8$^+$ T cell staining in lymph node (LN) sections. (a) Representative images of a whole LN section showing Gag p24 (yellow), BCL-6 (green) and FDCs (red). (b) Representative images showing Gag p24 (yellow) and FDC (red) co-staining. (c-d) Representative images of serial sections either showing (c) no co-staining between Gag p24 (yellow) and CD8$^+$ T cell marker (red) or (d) co-staining of Gag p24 (yellow or grey) with CD4$^+$ T cell (red) and FDC (red) markers. (e) Representative whole LN section with encircled GCs that are either Gag p24 positive (pos, yellow) or negative (neg, green) and a zoomed Gag p24 positive GC. Scale bar is 50 μm (f) The overall ratio of Gag p24 pos and neg GCs was compared across the groups. (g-h) The density of Gag p24 per GC in immunofluorescent images was computed using the nuclear segmentation method in TissueQuest™. Summary plots comparing results for (g) Fiebig I/II Tx, n=13; Fiebig III-V Tx, n=3; late Tx, n=9; and unTx, n=12; HIV-infected donors. (h) Aggregate results for donors Tx beyond 1 year (Fiebig I/II Tx, n=7; Fiebig III-V Tx, n=1; late Tx, n=5; and unTx, n=12). Each dot represents the density of Gag p24 per GC and the total number of GCs analysed per group is displayed. (i) Representative whole LN sections and zoomed Gag p24 positive regions. Three independent experiments were conducted with similar results. Error bars represent interquartile range. Data are presented as median ± interquartile range. All statistical tests were two-sided and adjusted *P* values from Dunn's multiple comparison's test are shown (g-h).

Supplementary Figure 2

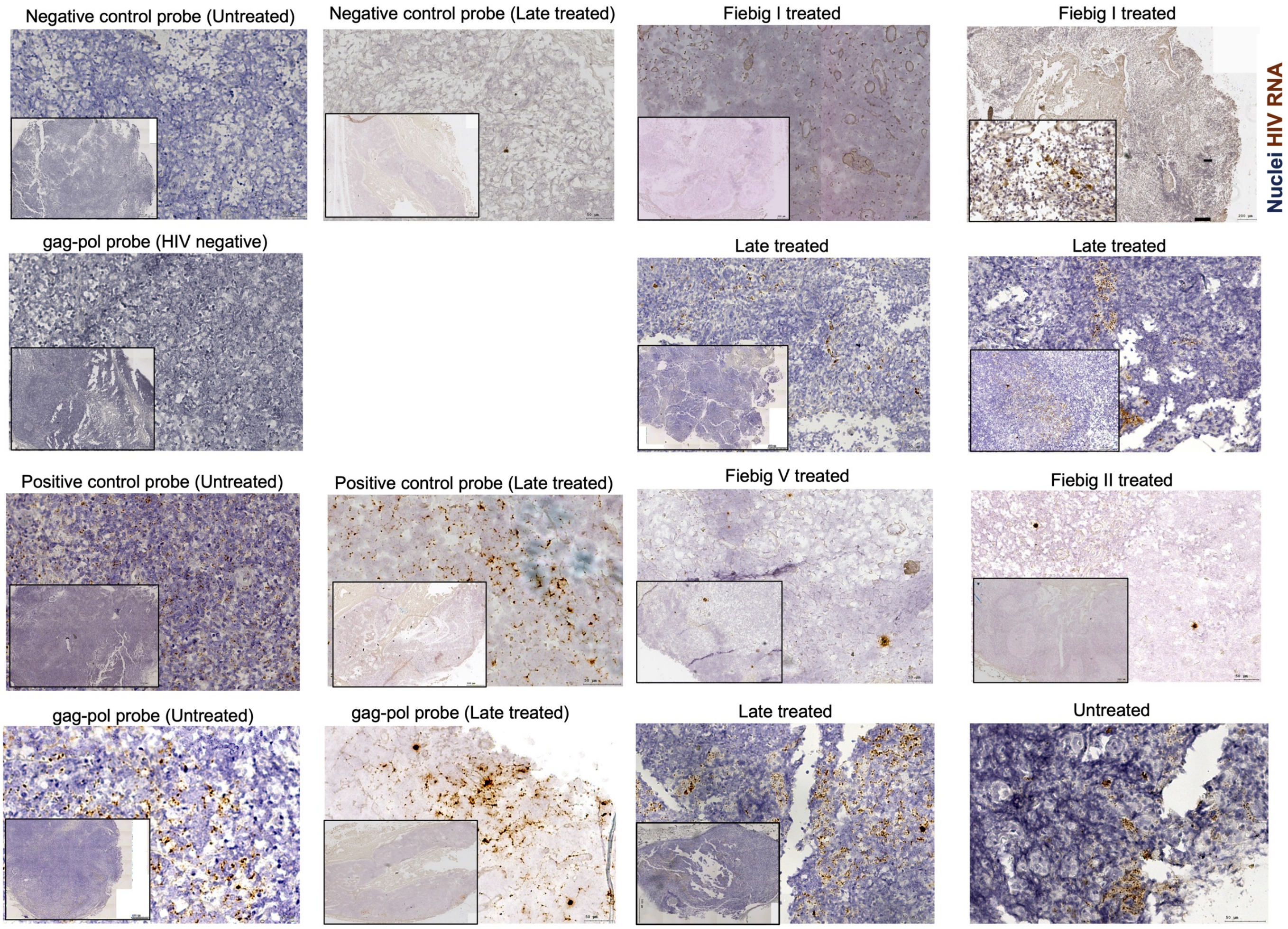

Supplementary Figure 2: Detection of RNA *in-situ* hybridization (ISH) images. Micrographs from *gag-pol* RNAscope ISH detected using 3,3'-diaminobenzidine as brown punctate dots with nuclei signals in blue. The specificity of staining was confirmed by using a probe for the bacterial gene *dapB* (negative control probe) or by using the *gag-pol* probe with samples for a HIV negative individual. In addition, a probe for human peptidylpropyl isomerase B was used as positive control. Inset is the full image of the magnified field of view. Three independent experiments were conducted with similar results.

Supplementary Figure 3

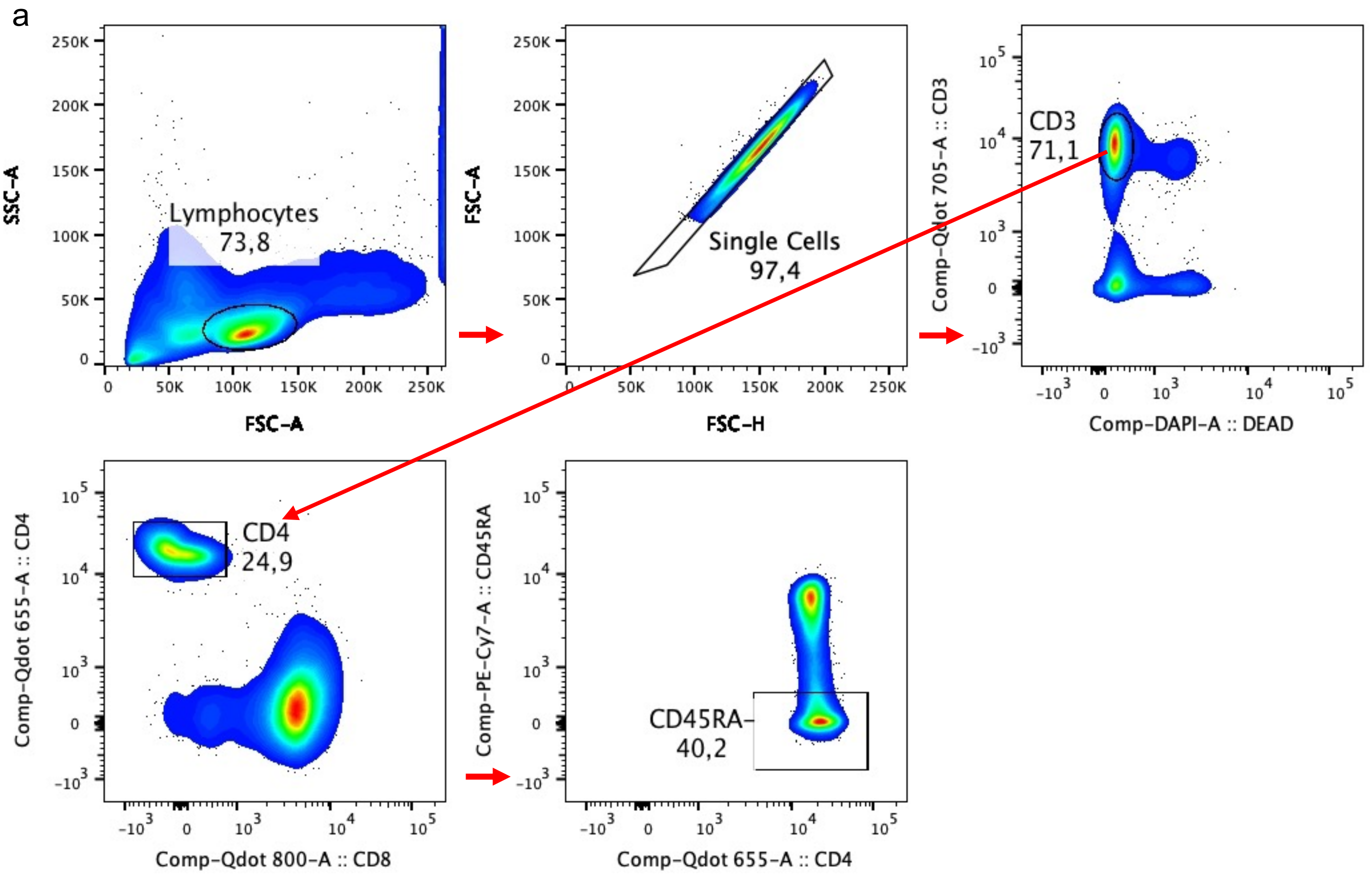

Supplementary Figure 3

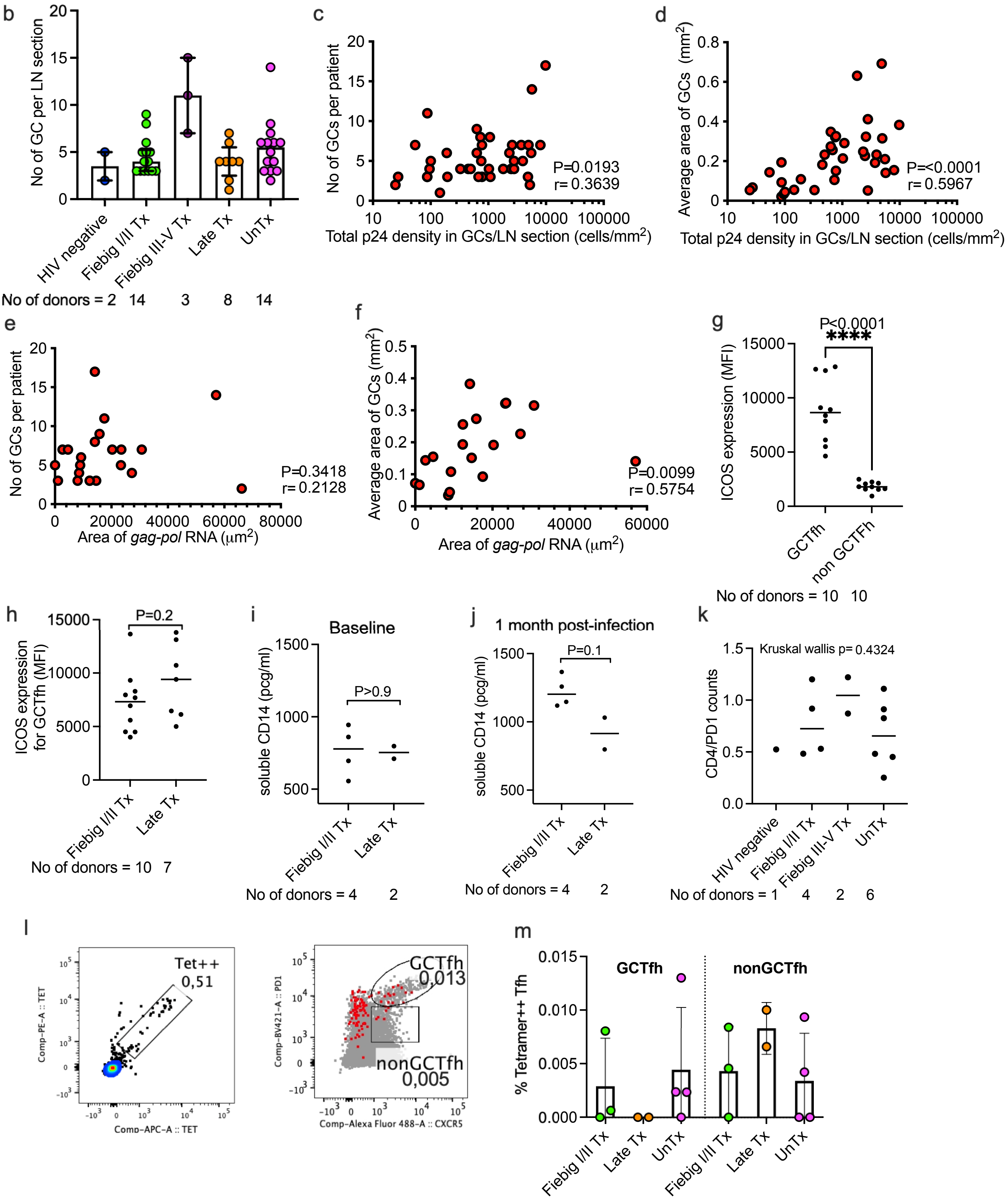

Supplementary Figure 3: The relationship between follicular characteristics (number and area of GCs) and HIV antigen persistence, as well as the quantitation of activated and HIV-specific Tfh cells. (a) Flow cytometry gating strategy for identifying GCTfh cells. The number of GCs (b) and the area density of GCTfh cells was computed from the analysis of IF microscopy images and correlated to the density of Gag p24 (c-d) and area of gag-pol RNA (e-f) for the same lymph nodes (LNs). (g-j) ICOS expression (MFI) by Tfh cells was measured in LN mononuclear cells using flow cytometry (g & h) and the amount of soluble CD14 in plasma samples (I & j) was measured at baseline and after 1 month of HIV infection. (k) Ratios of CD4 and PD-1 expressing cells within $BCL6^+$ GCs were quantified using image cytometry. (l) Gating strategy for identifying double-positive tetramer specific (Tet++) $CD4^+$ T cells and overlay plots of Tet++ $CD4^+$ T cells (red dots) on Tfh subsets with (m) aggregate data of Tet++ Tfh subsets across the groups. P values are from two-sided Mann-Whitney U tests (g-k & m). Spearman rho (*r*) and *P* values are reported (c-f). The median of each data set is indicated (g-k & m). Error bars represent interquartile range (b & m).

Supplementary Figure 4

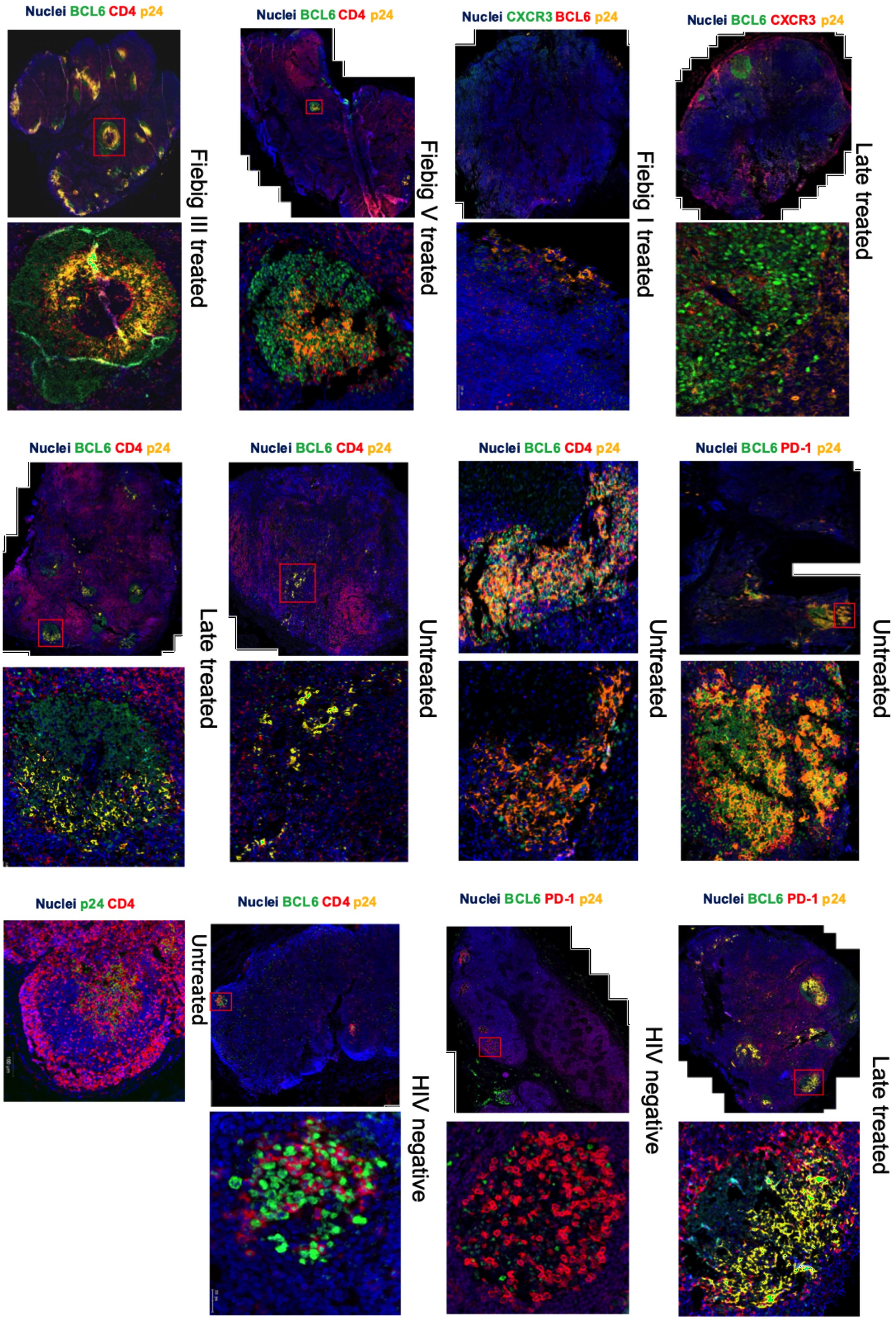

Supplementary Figure 4: HIV Gag p24 co-localizes with BCL-6 and CD4 or PD-1 markers in lymph node sections. Representative images of Gag p24 (yellow) and BCL-6 (green) and CD4 (red) or PD-1 (red) staining in LN sections of representative study participants. Three independent experiments were conducted with similar results.

Supplementary Figure 5

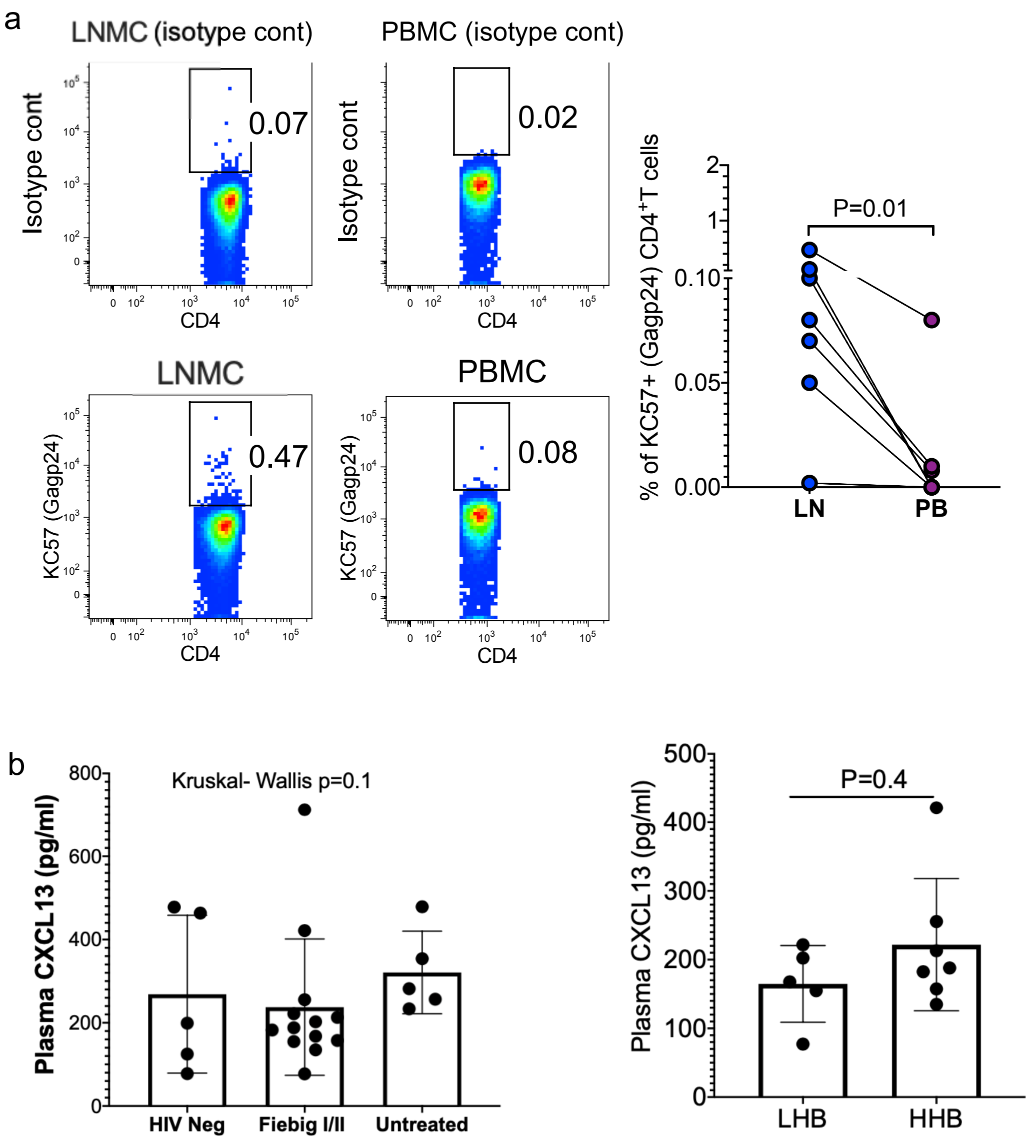

Supplementary Figure 5: Antigen persistence in treated HIV-1 infection (a) is not associated with plasma CXCL-13 levels (b). (a) Lymph node mononuclear cells (LNMCs) intracellularly stained with KC57 antibody (Gag p24). Flow plots and aggregate data show proportion of HIV-infected ($KC57^+$) $CD4^+$ T cells. (b) Plasma CXCL-13 levels in treated and untreated hyperacute HIV infection, and healthy controls as well as in high and low persistent HIV burden (HHB and LHB) in LNs during antiretroviral therapy. Statistical differences are calculated using two-sided Mann-Whitney U (a & b) and Kruskal-Wallis (b) tests. Error bars represent interquartile range (b). Data are presented as median ± interquartile range (b).
