# Supplementary material for: CD8 lymphocytes mitigate HIV-1 persistence in lymph node follicular helper T cells during hyperacute-treated infection
Source: Nat Commun. 2022 Jul 12;13:4041. doi: 10.1038/s41467-022-31692-8 (PMC9279299; doi:10.1038/s41467-022-31692-8)
Supplement: Supplementary file 3 — Reporting Summary [file 41467_2022_31692_MOESM3_ESM.pdf]

nature portfolio

Corresponding author(s): Prof Zaza M. Ndhlovu

Last updated by author(s): Jun 7, 2022

Reporting Summary

Nature Portfolio wishes to improve the reproducibility of the work that we publish. This form provides structure for consistency and transparency in reporting. For further information on Nature Portfolio policies, see our Editorial Policies and the Editorial Policy Checklist.

Statistics

For all statistical analyses, confirm that the following items are present in the figure legend, table legend, main text, or Methods section.

n/a	Confirmed	
☐	☒	The exact sample size (*n*) for each experimental group/condition, given as a discrete number and unit of measurement
☐	☒	A statement on whether measurements were taken from distinct samples or whether the same sample was measured repeatedly
☐	☒	The statistical test(s) used AND whether they are one- or two-sided<br>*Only common tests should be described solely by name; describe more complex techniques in the Methods section.*
☒	☐	A description of all covariates tested
☒	☐	A description of any assumptions or corrections, such as tests of normality and adjustment for multiple comparisons
☐	☒	A full description of the statistical parameters including central tendency (e.g. means) or other basic estimates (e.g. regression coefficient) AND variation (e.g. standard deviation) or associated estimates of uncertainty (e.g. confidence intervals)
☐	☒	For null hypothesis testing, the test statistic (e.g. *F*, *t*, *r*) with confidence intervals, effect sizes, degrees of freedom and *P* value noted<br>*Give P values as exact values whenever suitable.*
☒	☐	For Bayesian analysis, information on the choice of priors and Markov chain Monte Carlo settings
☒	☐	For hierarchical and complex designs, identification of the appropriate level for tests and full reporting of outcomes
☒	☐	Estimates of effect sizes (e.g. Cohen's *d*, Pearson's *r*), indicating how they were calculated

Our web collection on statistics for biologists contains articles on many of the points above.

Software and code

Policy information about availability of computer code

Data collection	Flowjo version 10.0.8, ImageJ version:2.0.0-rc-69/1.52p, TissueFAXS 6.129, TissueQuest 6.0.1.0136
Data analysis	Data were analyzed by GraphPad Prism version 7.0 for macOs

For manuscripts utilizing custom algorithms or software that are central to the research but not yet described in published literature, software must be made available to editors and reviewers. We strongly encourage code deposition in a community repository (e.g. GitHub). See the Nature Portfolio guidelines for submitting code & software for further information.

Data

Policy information about availability of data

All manuscripts must include a data availability statement. This statement should provide the following information, where applicable:

- Accession codes, unique identifiers, or web links for publicly available datasets
- A description of any restrictions on data availability
- For clinical datasets or third party data, please ensure that the statement adheres to our policy

The datasets generated in the study are provided in the supplementary information as source data file.

Field-specific reporting

Please select the one below that is the best fit for your research. If you are not sure, read the appropriate sections before making your selection.

☒ Life sciences ☐ Behavioural & social sciences ☐ Ecological, evolutionary & environmental sciences

For a reference copy of the document with all sections, see nature.com/documents/nr-reporting-summary-flat.pdf

Life sciences study design

All studies must disclose on these points even when the disclosure is negative.

Sample size	The study subjects and the sample size for this study were based on the available volunteers for the study and the samples acquired from the participants.
Data exclusions	Viremic individuals were excluded from the treated groups as pre-determined by the study design.
Replication	The study design encompassed biological replicates for all the different experiments. The details for each experiment are as follows: 3 independent immunofluorescence microscopy and RNAscope in situ hybridization experiments were performed successfully. Flow cytometry experiments were performed singly. RNA quantification was performed once and repeated only for randomly selected samples.
Randomization	Participants were allocated to groups based on their HIV statuses and their timing of antiretroviral treatment initiation.
Blinding	Investigators were blinded to clinical data when performing experiments.

Reporting for specific materials, systems and methods

We require information from authors about some types of materials, experimental systems and methods used in many studies. Here, indicate whether each material, system or method listed is relevant to your study. If you are not sure if a list item applies to your research, read the appropriate section before selecting a response.

Materials & experimental systems

n/a	Involved in the study	
☐	☒	Antibodies
☒	☐	Eukaryotic cell lines
☒	☐	Palaeontology and archaeology
☒	☐	Animals and other organisms
☐	☒	Human research participants
☐	☒	Clinical data
☒	☐	Dual use research of concern

Methods

n/a	Involved in the study	
☒	☐	ChIP-seq
☐	☒	Flow cytometry
☒	☐	MRI-based neuroimaging

Antibodies

Antibodies used	Antibodies for immunohistochemistry: anti-human BCL-6 [ready to use (RTU), (Clone PG-B6p), Cat# IR62561, Dako/Agilent Technologies, Glostrup, Denmark], CCR6 [1:400 dilution, (Clone R6H1), Cat# 14-1969-82, Thermofisher Scientific, Waltham MA, USA], CD4 [RTU, (Clone 4B12), Cat# R64961, Dako/Agilent Technologies], CXCR3 [1:400 dilution, (Clone 6H1L8), Cat# 702228, Thermo Fisher Scientific], CD8 [RTU, (Clone C8/144B), Cat# IR62361, Dako/Agilent Technologies], Follicular dendritic cells [1:25 dilution, (Clone CNA.42), Cat# M715701, Dako/Agilent Technologies] p24 [1:10 dilution, (Clone Kal-1), Cat# M085701, Dako/Agilent Technologies], and PD-1 [1:100 dilution, (Clone NAT105), Cat# AB52587-100, Abcam, Cambridge, MA, USA].<br>Antibodies for Flow cytometry: CD3 Brilliant Violet (BV) 711 (Cat# 317328, (Clone OKT3), BioLegend, San Diego, CA, USA), CD8 BV786 (Cat# 563823, (Clone RPA-T8,) BD Biosciences, San Jose, CA), CD4 BV650 [Cat# 563875, (clone SK3), BD Biosciences] CXCR5 Alexa Fluor (AF) 488 {Cat# 558112, (Clone RF8B2), (BD Biosciences), PD-1 BV421 [Cat# 329920, (Clone EH12 2H7), BioLegend], CCR6 Phycoerythrin [(PE) Cat# 353410, (Clone G034E3), BioLegend], CXCR3 BV605 [Cat# 353728, (Clone G025H7), BioLegend] and CD45RA PE-Cyanine (Cy)-7, [Cat# 304126, (Clone HI100), BioLegend], TNF-α A700 [Cat# 557996, (Clone MAb11), BD Biosciences], IFN γ PE-Cy7 [Cat# 506518, (Clone B27), BioLegend], streptavidin PE [Cat# SA10041, Thermofisher Scientific] and HIV Gag p24 RD1 [Cat# 6604667, (clone KC57), Beckman Coulter, Indianapolis, USA]. All flow cytometry antibodies were used at 1:40 dilution, except for HIV Gag p24 which was used at 1:200 dilution and IFN γ PE-Cy7 which was used at 1:10 dilution.
Validation	Antibodies used in the study are commercially available. They were used according to manufacturers instructions. Isotype controls were used to standardize staining protocols. Specifically, the ready-to-use mouse negative control antibody (Cat# IR75066, Agilent Technologies) or rabbit negative control antibody (Cat # IR60066, Agilent Technologies), were used to ascertain non-specific staining in the immunohistochemistry experiments. IgG1 isotype control (Cat# 400124, Biolegend), IgG1 isotype control (Cat# 51-35405X, BD Biosciences), or IgG2b isotype control (Cat# 553989, BD Biosciences), were used for flow cytometry staining validation.

Human research participants

Policy information about studies involving human research participants

Population characteristics	Our study cohort comprised of a total of 64 participants. 95% of which were females. They were grouped as follows: 13 HIV negative donors (HIV neg), 14 and 5 individuals who initiated ART either during hyperacute (Fiebig I/II Tx) or Fiebig III-V stages of HIV infection (Fiebig III-V Tx), 17 individuals who initiated treatment in Fiebig VI and beyond (late Tx) and 15 untreated individuals whose duration on infection is unknown (unTx). The ages for the participants in the HIV negative, Fiebig I/II Tx and Fiebig III-V Tx groups ranged from 18 to 26 years with a median age of 22 years. The participants in the late Tx and unTx groups were a little older, with a median of 24 years, ranging from 19 to 36 years for late Tx group and 18 to 49 years for the unTx group. Participants were reimbursed for their transport costs, with a sum of 32 USD on the day of the lymph node excision procedure and 10 USD at every other visit. All study participants provided informed consent prior to their participation in the study.
Recruitment	The participants were recruited from existing cohorts in Durban, South Africa, based on their provision of informed consent to donate a lymph node and a blood sample for our study. The recruitment process was independent (handled by the study nurse) of the subsequent experimental investigations conducted by the researchers. A potential bias in the recruitment process may be the willingness of individuals to undergo the more invasive lymph node collection process. But this does not have a clear impact on the biological outcomes of the investigations. Sample availability limited some investigations to certain participants and this was based on the size of the lymph nodes.
Ethics oversight	Ethical approval for the study was granted by the University of KwaZulu-Natal Biomedical Research Ethics Committee (protocol number BF298/14) and the Institutional Review Board of Massachusetts General Hospital (protocol number 2015-P001018).

Note that full information on the approval of the study protocol must also be provided in the manuscript.

Clinical data

Policy information about clinical studies

All manuscripts should comply with the ICMJE guidelines for publication of clinical research and a completed CONSORT checklist must be included with all submissions.

Clinical trial registration	*Provide the trial registration number from ClinicalTrials.gov or an equivalent agency.*
Study protocol	*Note where the full trial protocol can be accessed OR if not available, explain why.*
Data collection	*Describe the settings and locales of data collection, noting the time periods of recruitment and data collection.*
Outcomes	*Describe how you pre-defined primary and secondary outcome measures and how you assessed these measures.*

Flow Cytometry

Plots

Confirm that:

- [x] The axis labels state the marker and fluorochrome used (e.g. CD4-FITC).
- [x] The axis scales are clearly visible. Include numbers along axes only for bottom left plot of group (a 'group' is an analysis of identical markers).
- [x] All plots are contour plots with outliers or pseudocolor plots.
- [x] A numerical value for number of cells or percentage (with statistics) is provided.

Methodology

Sample preparation	Sample preparation is detailed in the methods section of the manuscript
Instrument	LSRFortessa and FACS Aria Fusion (BD Biosciences)
Software	FACSDiva™ software (BD Biosciences) was used for acquisition and FlowJo version 10.0.8 (Flowjo, LLC, Ashland, Oregon), was used for analysis.
Cell population abundance	The cell-counts for post-sort fractions were usually in the range of 50,000 to 100,000 cells and the purity was verified by flow cytometry. The purity was usually >95%.

Gating strategy

Lymphocytes were gated based on the distribution of the forward scatter (FSC) area and side scatter (SSC) area. Doublets were excluded in the single cell gating depending on the height (FSC-H) and area (FSC-A) of the forward scatter. The CD4 population gate originated from the live CD3+ cells and non naive CD4 T cells were identified using CD45RA negative gate. Subsequently, GCTfh cells were identified by the double expression of CXCR5 and PD-1 markers. For the identification of CD8 T cells, the CD8 gate originated from the live CD3+ gate. Fluorescence minus one (fmo) controls were used to define the correct positioning of the gates.

☒ Tick this box to confirm that a figure exemplifying the gating strategy is provided in the Supplementary Information.
